# Mucinous tubular and spindle cell carcinoma of the kidney

**DOI:** 10.4322/acr.2023.415

**Published:** 2023-01-06

**Authors:** Neha Bhardwaj, Mayur Parkhi, Debajyoti Chatterjee, Shrawan Kumar Singh

**Affiliations:** 1 Post Graduate Institute and Medical Education and Research, Department of Histopathology, Chandigarh, India; 2 Post Graduate Institute and Medical Education and Research, Department of Urology, Chandigarh, India

Mucinous tubular and spindle cell carcinoma (MTSCC) is a unique neoplasm attributing to less than 1% of all renal cell carcinoma (RCC). The median age is 6th decade with a significant female preponderance.^1^ These tumors classically consist of tightly packed, elongated, and anastomosing tubules which merge with bland spindle cells in a myxoid stroma in variable proportions. Tubules with tufting or small papillae and foci of foamy macrophages may occur. The tumor cells are low-grade and may show clear cytoplasm or oncocytic changes with rare mitosis. Immunohistochemically, PAX8, CK7, AMACR, and CD10 are positive in this tumor.^1^^,^ 2 Though it is a morphological diagnosis, it can be challenging to differentiate from a solid variant of papillary RCC, sarcomatoid RCC, or myoid-predominant angiomyolipoma. Immunohistochemistry may be of little help due to overlapping profiles.^3^ Copy number analyses can help establish a diagnosis in challenging cases or core biopsies since these are associated with multiple chromosomal losses involving chromosomes 1, 4, 6, 8, 9, 13, 14, 15, and 22. Novel biomarkers like VSTM2A overexpression are also emerging, which can be detected by RNA in-situ hybridization. Recurrent Hippo pathway aberrations have been defined as the molecular signature of MTSCC with increased nuclear YAP1 protein expression.^4^ Adverse features such as necrosis, solid growth, single file infiltration, sarcomatoid transformation, lymphovascular invasion, and increased mitoses are indicators of metastatic disease. Although it has indolent behavior, rare cases with classic morphology have been seen to develop metastases.^5^^-^ 6

**Figure 1:**
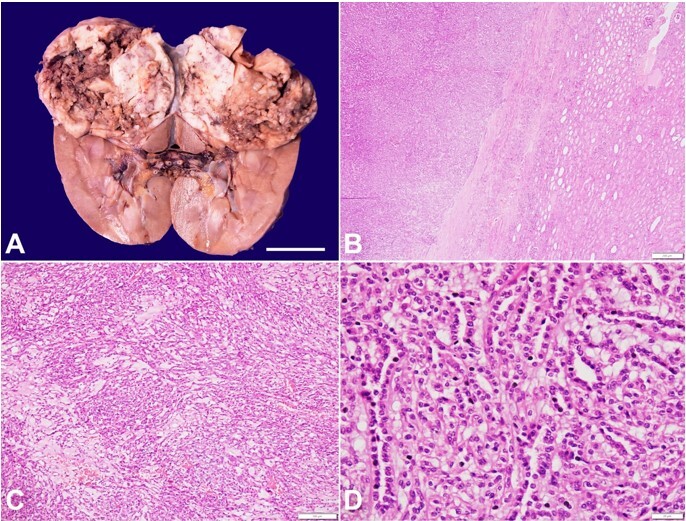
**A -** gross nephrectomy specimen showing a large, well-demarcated tumor involving the upper pole of the kidney measuring 6.9x6.5x5.5cm. The cut surface is variegated and appears grey-white and firm, along with intervening mucinous and hemorrhagic areas (scale bar = 5 cm); **B -** microscopically, the tumor is well-circumscribed with sharp demarcation from adjacent normal renal parenchyma (H&E; 40x); **C -** the dominant epithelial component is seen against the background stroma containing significant extracellular mucin (H&E; 100x); **D -** higher magnification showing tightly packed anastomosing tubules lined by low-grade cuboidal cells with cytoplasmic vacuoles against mucinous background (H&E; 400x).

We describe gross and histopathological findings of mucinous tubular and spindle cell carcinoma in a 65-year-old male patient. He presented with right abdominal pain for one month. The pain was dull and mild to moderate in intensity. On contrast-enhanced ultrasound, a well-defined, smoothly marginated heterogeneous hyperechoic lesion measuring 5.5x6cm was noted at the upper pole of the right kidney. The lesion was hyper enhancing compared to the rest of the renal parenchyma. Based on the radiological findings, possibilities of chromophobe RCC and oncocytoma were considered. The abdominal computed tomography (CT) revealed a hyperdense lesion in the upper pole of the right kidney with a relatively well-defined margin measuring 63x65x69mm with no evidence of significant post-contrast enhancement in the either arterial, venous or delayed phase. The patient underwent a right radical nephrectomy. On gross examination, a well-encapsulated mass was seen wholly occupying the upper pole of the kidney measuring 6.9x6.5x5.5cm. The cut surface was variegated with grey-white, firm areas admixed with mucinous and hemorrhagic foci (Figure 1A). No capsular breach or perinephric fat extension was noted. The renal pelvis, sinus, and renal vessels were free of tumor.

Microscopically, the tumor was well-demarcated from the adjacent native renal parenchyma (Figure 1B). It was composed of the dominant epithelial element against the background of extracellular mucinous stroma (Figure 1C). The epithelial component contained tightly packed, elongated, tufted, and anastomosing tubules lined by low-grade cuboidal cells and are seen merging with bland spindle cells (Figure 1D). The nuclei display fine vesicular chromatin, inconspicuous nucleoli, and moderate cytoplasm with vacuolations. Mitotic figures are infrequent (<1/10HPF). No necrosis, lymphovascular invasion, or high-grade transformation was found. However, foamy macrophage collections and lymphoid aggregates are admixed. Cholesterol clefts with focal foreign body giant cell response indicated the long-standing nature of the tumor. Based on the gross and microscopy findings, the diagnosis of mucinous tubular and spindle cell carcinoma [(pT1bpNx; American Joint Committee on Cancer (AJCC) staging manual; 8th edition)] was rendered.
